# The Deposition and Properties of Titanium Films Prepared by High Power Pulsed Magnetron Sputtering

**DOI:** 10.3390/ma16237294

**Published:** 2023-11-23

**Authors:** Quanxin Jiang, Donglin Ma, Yantao Li, Changzi Chen

**Affiliations:** 1 Jingchu University of Technology, Jingmen 448000, China; a9165@126.com; 2 Chengdu Normal University, Chengdu 611130, China; mdl208115@163.com; 3 Mianyang Normal University, Mianyang 621000, China; liyantao1992@126.com

**Keywords:** high-power pulsed magnetron sputtering technology (HPPMS/HiPIMS), Ti film, stress, corrosion resistance, friction and wear resistance

## Abstract

Titanium thin films are particularly important as electrode layers, barrier layers, or intermediate buffer layers in the semiconductor industry. In order to improve the quality of Ti thin films and the adhesion and diffraction abilities of irregular parts, this paper used high-power pulsed magnetron sputtering (HPPMS/HiPIMS) to prepare titanium thin films. The effects of different trigger voltages (700 V, 800 V, and 900 V) on plasma properties were studied, and the microstructure, mechanical properties and corrosion resistance of the films were also studied. The results showed that as the voltage increased, the grain size of the thin films gradually increased. The residual stress of the titanium films changed from compressive stress (−333 MPa) to tensile stress (55 MPa) and then to low compressive stress (−178 MPa). The hardness values were 13 GPa, 9.45 GPa and 6.62 GPa, respectively. The wear resistance of the films gradually decreased, while the toughness gradually increased. The corrosion resistance of the films decreased as well.

## 1. Introduction

With the rapid rise of the semiconductor industry, magnetron sputtering technology has been widely used to deposit metal electrode layers in integrated circuit transistors. As an electrode layer material, metal Ti thin film can be used as an adhesive layer to reduce contact resistance, or as a barrier layer to block the diffusion between the silicon substrate and the connecting line [1]. At the same time, it can serve as a buffer layer to reduce the stress between the coating and the silicon substrate, and improve the adhesion between the film and the substrate [2,3]. However, the deposition sites of semiconductor materials are often irregular parts such as grooves. Conventional DC magnetron sputtering techniques, due to their poor diffraction ability, result in poor film quality. In order to improve the uniformity of thin film deposition, researchers proposed the use of high-power pulsed magnetron sputtering (HPPMS) technology to prepare Ti thin films in grooves [4]. The films exhibit 60% equiaxed crystals, increasing their density. Due to the high metal ionization rate (20–70%) of HPPMS technology [5], the ionization rate of Ti can reach as high as 90% [6], greatly improving the quality of thin films, improving the uniformity of irregular parts [7], and enhancing the film–substrate adhesion force [8]. In order to obtain a higher ionization rate, researchers studied the effects of different working pressures [9], trigger voltages [10], peak currents [11], and magnetic field intensities [12] on the ionization rate. The study found that the higher the peak current, the higher the ionization rate. As the body of research on ionization rates continues to grow [13,14,15], most researchers are also gradually paying attention to the effects of production processes, ionization rates, and film performances. Research has found that Ti film transitions from compressive stress to tensile stress [16], which affects the performance of the film. However, the relationship between plasma properties and film properties remains complex. In order to study the plasma properties and mechanical properties of Ti thin films deposited via HPPMS under different trigger voltages, this paper deposited Ti thin films on silicon wafers by varying the trigger voltages (700 V, 800 V, and 900 V), measuring the ion–atom ratio near the substrate using spectroscopy, and studying the effect of ionization degree on the stress of the thin film, thereby studying the mechanical properties and corrosion resistance of the thin films.

## 2. Materials and Methods

### 2.1. Sample Preparation

Titanium films were deposited on Si (100) and 316 L stainless steel substrates using a non-equilibrium magnetron sputtering instrument [17]. The vacuum chamber size was φ500 × 500, the vacuum chamber pressure reached 1.0 × 10^−5^ Pa, the argon (99.999%) flow rate was 60 sccm, the target–base distance was 60 mm, and the target and sample were cleaned with Ar ions for 5 min and 10 min, respectively. In the test, a HPPMS power supply (HPS-450D, Chengdu Pulse tech Electrical, Chengdu, China) was used to power the target. A Ti target (99.99%) was selected as the sputtering target. The specific preparation process and the process parameters of the HPPMS deposition of Ti films under the different trigger voltages are shown in Table 1.

### 2.2. Film Characterization and Testing

In order to study the discharge characteristics of the high-power pulsed magnetron sputtering power supply, the voltage and current in the discharge process were monitored using a voltage probe (Tektronix, p-5100, Beaverton, OR, USA) and a current transformer (Pearson, 411, London, UK), and recorded using an oscilloscope (Tektronix, tds-220). Emission spectrometers (avantes, avaspec-2048-7-usb2) were used to monitor the plasma composition 10 mm in front of the substrate. The grating specification was configured with 2400 pieces/mm, the slit width was 10 μm, and the wavelength range was 200–1100 nm. The thickness and residual stress of the films were measured with a step meter (ambios xp-2). The structure of the silicon-based Ti films was characterized using X-ray diffractometry (XRD, Philips X’pert, and Netherlands) and transmission electron microscopy (TEM). The X-ray diffractometer used a copper target (λ = 1.54060 Ω). The X-ray tube voltage was 40 kV and the current was 40 mA. The surface morphology and cross-sectional morphology of the titanium films were observed using a field emission scanning electron microscope (SEM). The hardness and toughness of the films were evaluated using a nano-hardness tester (Anton Paar CSM, Buchs, Switzerland), and the hardness and elastic modulus of the films were characterized. The wear resistance of the Ti films was evaluated using a ball–disc reciprocating friction machine (CSEM, Neuchâtel, Switzerland) at 55% RH relative humidity and a temperature of (12 ± 1) °C. The grinding pair was a GCr15 ball (diameter 6 mm) with a load of 0.5 N, 3.77 cm/s, and a wear of 1000 RPM (reciprocating stroke 40 m). The depth and morphology of wear marks were observed using a step meter (ambios xp-2) and an optical microscope (sdptop mx6r forward microscope). An electrochemical workstation (Shanghai Chenhua chi660i series) was used to evaluate the corrosion resistance of the titanium films. The test conditions of the polarization curve were a solution system of 3.5%NaCl solution, a scanning rate was 0.005 V/s, and a test potential was −0.8–2 V [18].

## 3. Results

### 3.1. Target Discharge and Plasma Characteristics

**Figure 1:**
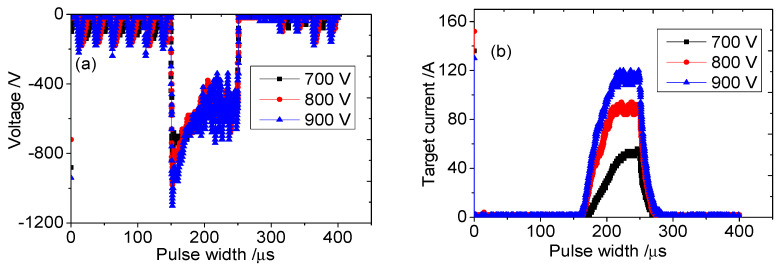
(**a**) Discharge voltage and (**b**) current of the HPPMS-sputtered Ti target under different trigger voltages.

Figure 1 shows the time variation curves of the Ti target discharge voltage and discharge current under different trigger voltages. As can be seen from the figure, when the trigger voltages were 700 V, 800 V, and 900 V, the peak currents of the target were 60 A, 100 A, and 120 A. The peak current increased with the increase in sputtering voltage. As the sputtering voltage increases, a large number of argon ions bombard the target material, increasing the probability of a large number of electrons near the target colliding with neutral particles and particles. This generates a large number of secondary electrons, which can effectively ionize the sputtered atoms, further increasing the probability of collisions between electrons and neutral particles. The discharge current of the target is composed of various ion currents [13,15], resulting in an increase in the peak current. When the sputtering voltage is 900 V, the peak current slope is steepest, and when the sputtering voltage is 700 V, the peak current slope is shallowest. This shows that as the voltage increases, the increase rate of peak current under different voltages also becomes faster. This is because the electron density in the ionization region is positively correlated with the slope of the rising edge of the current waveform [19]. When the sputtering voltage increases, a large number of secondary electrons are generated near the target, resulting in an increase in current and speed.

**Figure 2:**
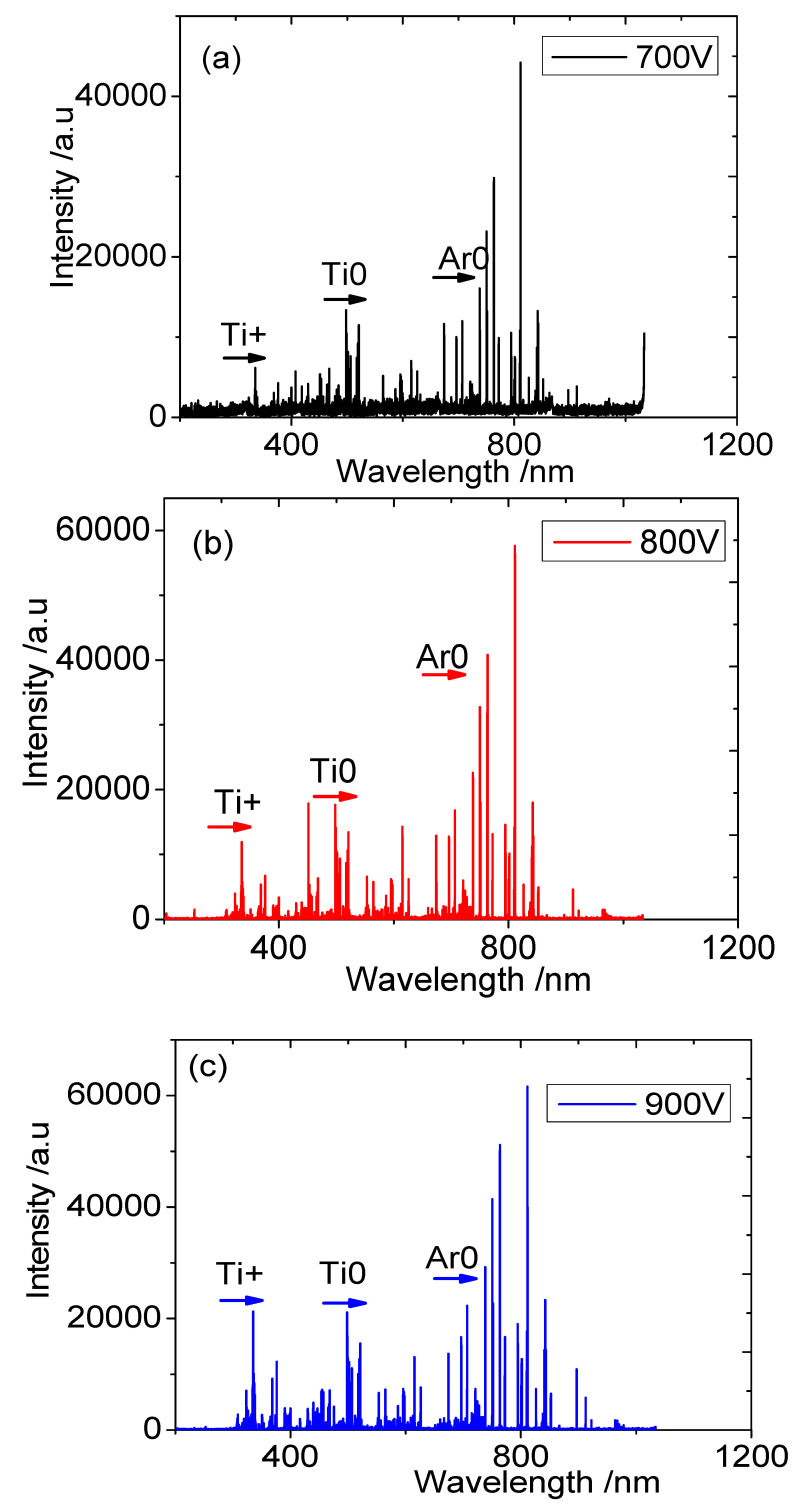

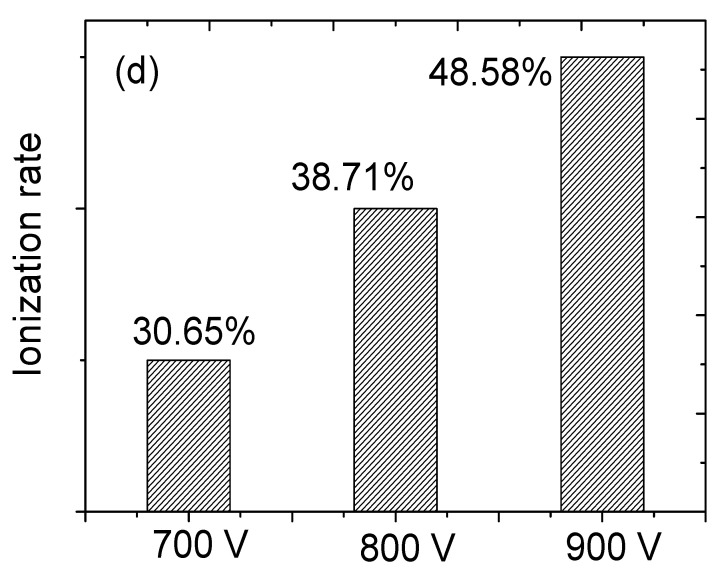
Emission spectral intensity and ionization rates of plasma components under different trigger voltages: (**a**) 700 V, (**b**) 800 V, (**c**) 900 V, and (**d**) the ionization rate.

Figure 2 shows the plasma emission spectral intensity values and the ion-to-atom ratios of Ti in front of the substrate under the different trigger voltages when Ti film is deposited using HPPMS technology. The plasma emission spectra of the first 10 mm of the substrate are mainly composed of Ti^+^, Ti^0^, and Ar^0^. It can be seen from Figure 2a–c that with the increase in trigger voltage, the emission intensity of each particle also increases gradually. The spectral intensity of the six main characteristic peaks, whose wavelengths were Ti^+^ (334.83, 334.89, 336.09 nm) and Ti^0^ (498.2, 499.13, 499.97 nm), was selected to calculate the ion–atom ratio [20]. As shown in Figure 2d, the ion–atom ratios of Ti were 30%, 38%, and 48%, respectively. This shows that the ion–atom ratio of Ti increases gradually with the increase in trigger voltage.

The ionization mechanisms of HPPMS are mainly the collision of electrons with neutral atoms, Penning ionization, and charge conversion [13]. Penning ionization is a process in which electrons move in a spiral shape under the joint action of electric and magnetic fields, and a large number of electrons rotate into the vicinity of the anode in the form of a roller line under the bondage of magnetic fields, forming an electron cloud layer. Through the collision of electrons with metal atoms, the collision of metastable argon atoms with metal atoms, and the collision of argon particles with argon ions and the Ti target, a large amount of Ti^+^ is generated in the ionization region. With the increase in trigger voltage, a large number of secondary electrons are generated near the target, which increases the probability of collision ionization and causes the ratio of ions to atoms to rise.

### 3.2. Microstructure and Composition

**Figure 3:**
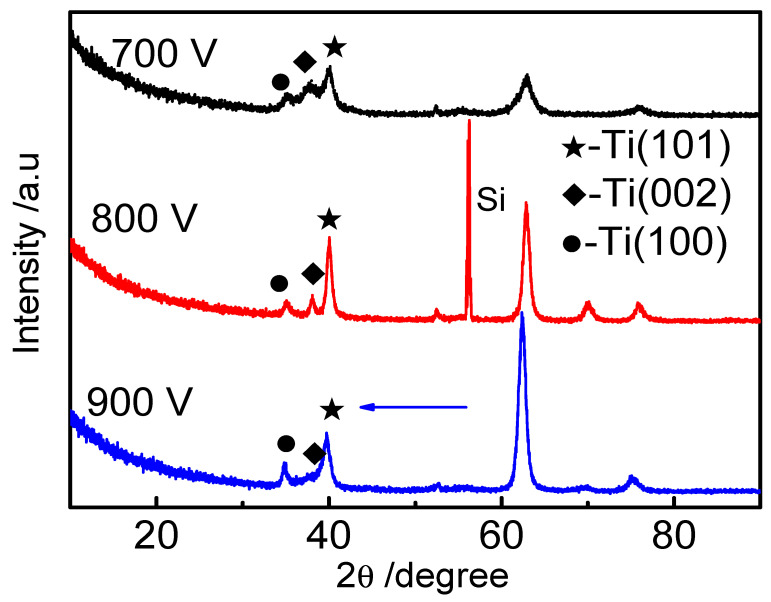
XRD patterns of Ti thin films deposited by HPPMS under different trigger voltages.

**Figure 4:**
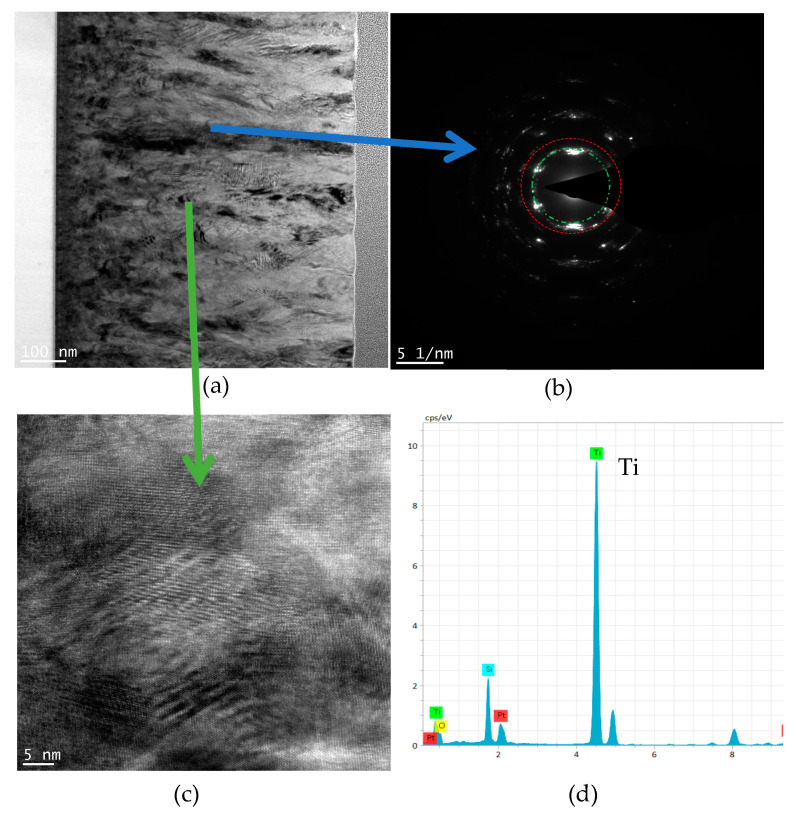
Cross-sectional TEM pattern of Ti thin film deposited by HPPMS at a trigger voltage of 900 V. (**a**) Cross-sectional morphology, (**b**) selected area electron diffraction (SAED) pattern, (**c**) high-resolution spectrum of cross-sectional, and (**d**) selected-area EDS spectrum.

Figure 3 shows the XRD pattern of Ti films deposited via HPPMS at different trigger voltages. It can be seen from the figure that Ti (100), Ti (002), and Ti (101) diffraction peaks appear in Ti films at the different trigger voltages. Figure 4a shows the TEM spectrum of the Ti film deposited via HPPMS at 900 V, the electron diffraction shows obvious Ti (101) structure in Figure 4b, the high-resolution spectrum shows obvious crystal structure in Figure 4c, and Figure 4d shows that the film was mainly Ti film with a small amount of oxygen, which was due to the presence of a small amount of oxygen in the initial stage of the film. Oxygen was gradually expelled.

**Figure 5:**
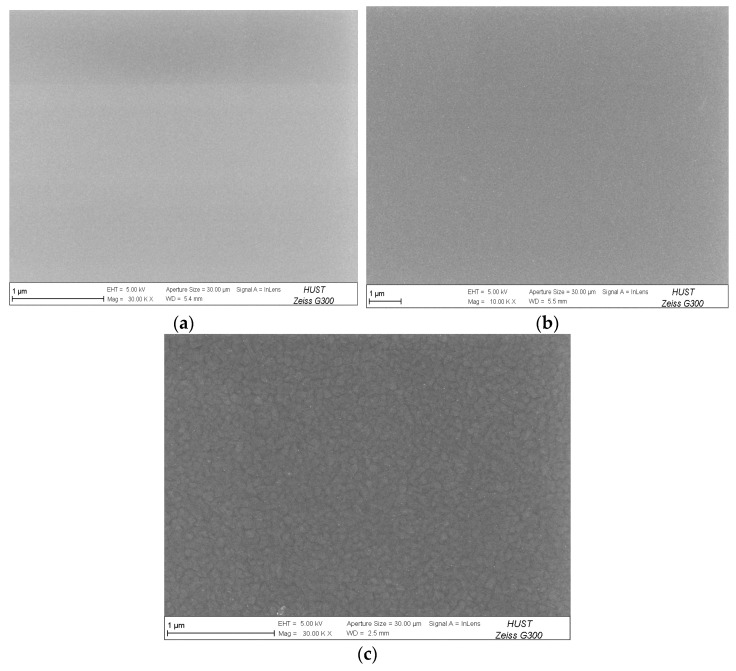
Surface morphology of Ti thin films deposited by HPPMS under different trigger voltages: (**a**) 700 V, (**b**) 800 V, and (**c**) 900 V.

**Figure 6:**
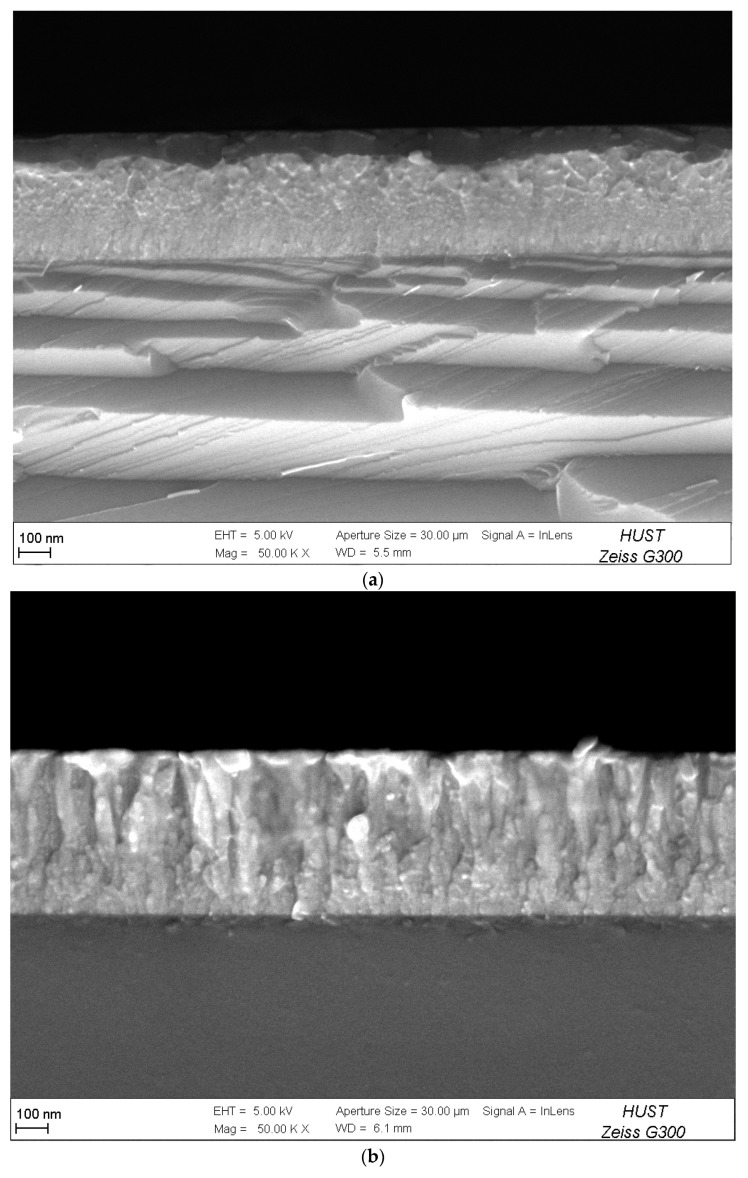

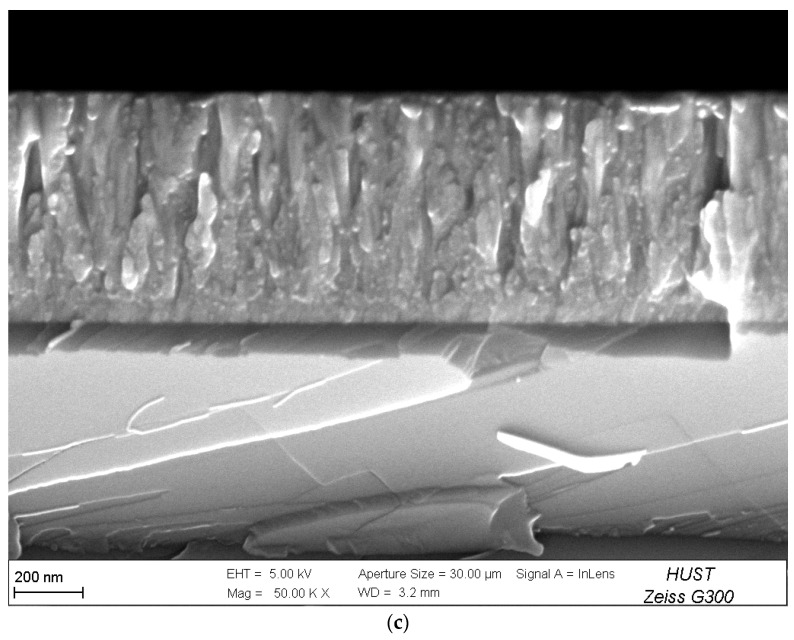
Cross-sectional morphology of Ti thin films deposited via HPPMS under different trigger voltages: (**a**) 700 V, (**b**) 800 V, and (**c**) 900 V.

Figure 5 and Figure 6 show the surface morphology and cross-sectional morphology of Ti films under different trigger voltages, respectively. It can be seen from Figure 5 that with the increase in trigger voltage, the ion–atom ratio increases, and the surfaces of the Ti films become smoother and less dense, which is due to the migration and growth of particles at deposition temperature [21].

As can be seen from Figure 6, when the trigger voltage is 700 V, the ionic atoms of Ti are relatively small, the interface is mainly columnar crystal growth, the top spacing is relatively regular, and the surface of the film is defective from the cross section. In general, the surface is smooth and dense. When the trigger voltage is 900 V (Figure 6c), the slender columns of the Ti film are further reduced.

### 3.3. Mechanical Properties of the Film

**Figure 7:**
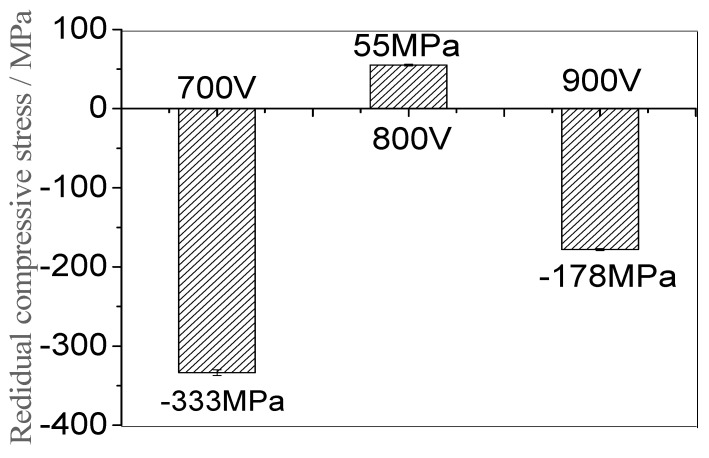
Residual stress of Ti thin films deposited by HPPMS under different trigger voltages.

Figure 7 shows how the curvature method was used to measure and record the curvature change of the film deposited on the Si (100) matrix, and the residual stress of the Ti films under the different voltages was calculated [22]. The residual stress of the film can be generated using the deposition process of the film, or using the mismatch between the film and the substrate during the deposition temperature cooling process (the thermal expansion coefficient is different). The residual stress of the film can be divided into internal stress, thermal stress and external stress. As can be seen from Figure 7, as the voltage increased from 700 V to 800 V, the residual compressive stress of the Ti films continued to decrease from −333 MPa to 55 MPa, transforming into tensile stress. This is because the Ti atomic ratio increases with the increase in voltage, resulting in a continuous increase in the substrate surface temperature, and thus an increase in atomic mobility. Additionally, film recrystallization releases compressive stress. When the voltage was increased to 900 V, the residual stress of the film changed from a tensile stress of 55 MPa to a compressive stress of −178 MPa. This may be because during the film growth process, when the trigger voltage was increased to 900 V, a large number of electrons impacted the argon particles, which enhanced the bombardment effect of the high-energy gas particles, resulting in an increase in the residual compressive stress of the film. It can also be seen from the XRD that the overall structure shifted to the left [23].

**Figure 8:**
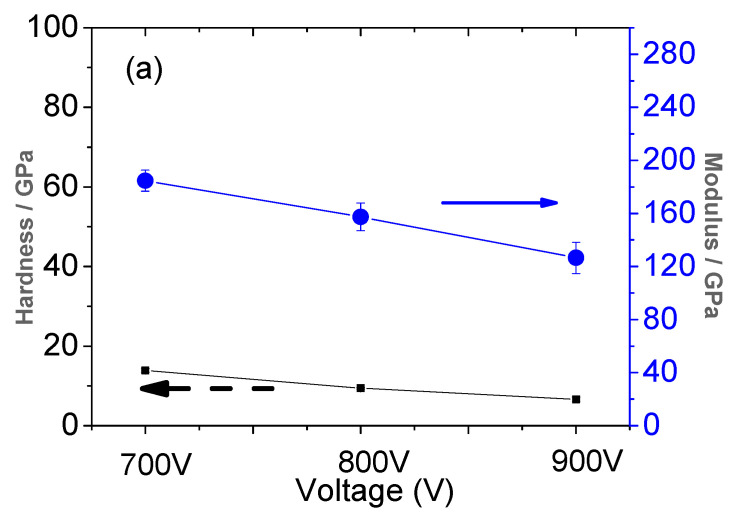

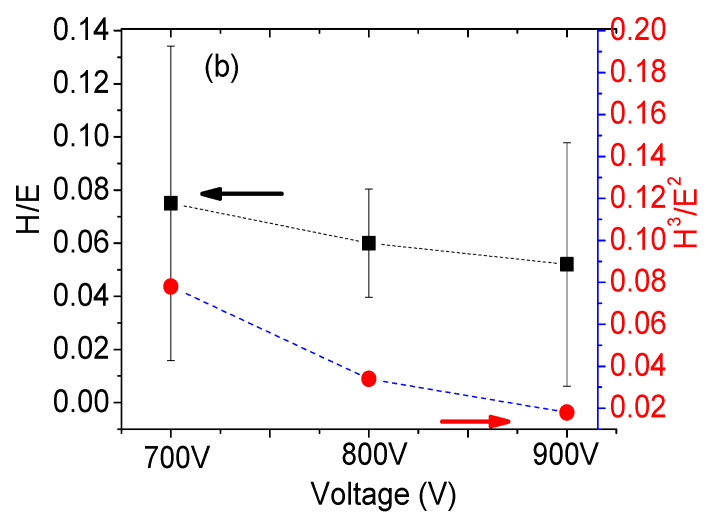
(**a**) Hardness H and modulus E. (**b**) H/E and H^3^/E^2^ values of Ti thin films deposited by HPPMS under different trigger voltages.

A nano-hardness method was used to measure the hardness of Ti films prepared under different trigger voltages. The maximum load was 20 mN and the loading and unloading rate was 40 nm/s. The hardness and elastic modulus of Ti films prepared under different voltages were obtained, as shown in Figure 8a,b which show the ratio of hardness to modulus under the different trigger voltages. In Figure 8a, the left side is the hardness of the film, and the right side is the elastic modulus of the film. When the voltage was 700 V, the hardness of the film was 13 GPa. When the voltage was raised to 800 V, the hardness of the film decreased to 9.45 GPa. When the voltage was raised to 900 V, the hardness of the film continued to decrease to 6.62 GPa, with a smaller reduction. This may be because the surface hardness of the metal film was affected by the residual stress, and the residual compressive stress made the film more resistant to the penetration of the indenter. With the increase in voltage, the residual compressive stress of the film would reduce the surface hardness of the film, and when the voltage increased from 800 V to 900 V, the residual compressive stress would slightly increase (Figure 8). At this time, the surface hardness of the film would be reduced.

The elastic modulus decreased from 180 GPa to 160 GPa and then from 160 GPa to 120 GPa with the increase in voltage, which indicates that the elastic modulus decreases with the increase in voltage and that the reduction amplitude is small first and then large. In other words, the elastic modulus is closely related to the internal stress of the film. When the voltage increases, the compressive stress generated by the film decreases, and so the elastic modulus of the film also decreases. When the voltage was increased to 900 V, the compressive stress of the film was slightly increased, but the elastic modulus was greatly decreased, which indicates that elastic modulus is not only affected by the internal stress of the film, but also affected by other factors. This may be because when the sputtering voltage increases to 900 V, the depth of penetration by ionized high-energy ions in the target increases and the energy loss increases, making it more difficult for the target atoms to escape. Additionally, the film deposition rate decreases, resulting in the film thickness of the film being reduced and the elastic modulus being greatly reduced. H^3^/E^2^ in Figure 8b represents the ratio of film hardness (H) to elastic modulus (E), which describes the limit of elastic strain. H^3^/E^2^ represents a strong indicator of the film’s resistance to plastic deformation after contact with an external load [24]. It can be seen from Figure 8b that the ratios of H^3^/E^2^ at 700 V, 800 V and 900 V are 0.075, 0.06 and 0.052, respectively, indicating that when the voltage increases, the elastic strain limit of the film tends to decrease and the toughness increases. This may be because when the voltage rises, the residual compressive stress generated by the film decreases, resulting in the hardness of the film decreasing, and the brittleness also decreasing, so the toughness increases and the ability of the film to recover after deformation is also reduced. For the film, when the voltage changed from 800 V to 900 V, the ratio increased slightly, which may be related to film thickness.

The ratios of H^3^/E^2^ at the different voltages were 0.078, 0.034, and 0.018, respectively. This indicates that the plastic deformation resistance of the thin films decreases with the increase in the sputtering trigger voltage.

### 3.4. Tribological Properties of Thin Films

**Figure 9:**
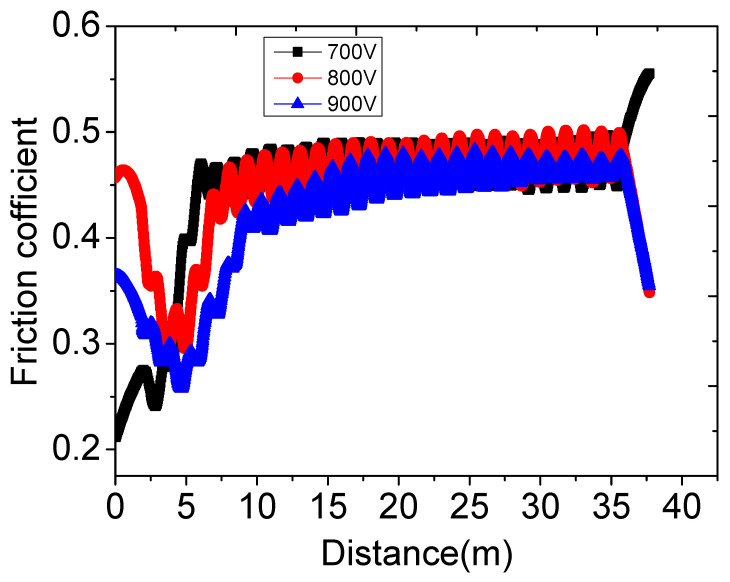
Friction coefficient of Ti thin films deposited via HPPMS under different trigger voltages.

**Figure 10:**
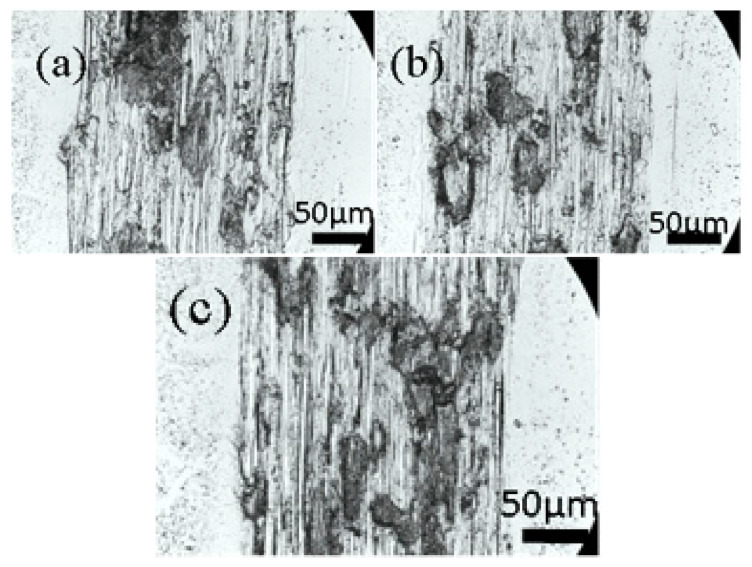
Wear scar morphology of Ti thin films deposited via HPPMS under different trigger voltages: (**a**) 700 V, (**b**) 800 V, and (**c**) 900 V.

The wear resistance of Ti films was evaluated using a ball–disc reciprocating friction machine at a relative humidity of about 55% RH and a temperature of 12 ± 1 °C. A GCr15 ball (diameter 6 mm) was selected for the grinding pair, the load was 0.5 N, 3.77 cm/s, and the wear was 1000 RPM. Figure 9 shows the friction coefficient of Ti films deposited under the different trigger voltages, and Figure 10 shows the wear morphology of Ti films deposited under the different trigger voltages. It can be seen from Figure 9 that when the trigger voltage is 900 V, the friction coefficient of the Ti film is small. As can be seen in Figure 10, the film experienced different degrees of wear and accumulation after 1000 revolutions and 40 m of reciprocating stroke.

**Figure 11:**
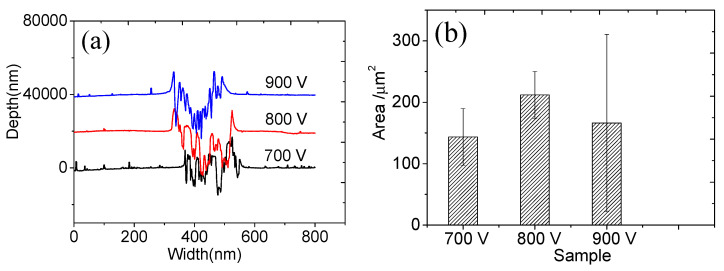
(**a**) Depth of wear marks and (**b**) cross-sectional area of wear marks in Ti thin films deposited via HPPMS under different trigger voltages.

The width and depth of the swept surface of the wear marks were mapped using the step meter, as shown in Figure 11a. The cross-sectional area of the wear marks was obtained by integrating Figure 11a, as shown in Figure 11b. When the sputtering voltage was 700 V or 900 V, the cross-sectional area of the wear mark was small, which can also be seen from the friction coefficient in Figure 9. H^3^/E^2^ can also be used to measure the wear resistance of the film [24], which is consistent with the results shown in Figure 11b. The lower the sputtering voltage, the greater the H^3^/E^2^ ratio, the stronger the film’s resistance to plastic deformation, and the more wear-resistant the film.

### 3.5. Film Corrosion Resistance

**Figure 12:**
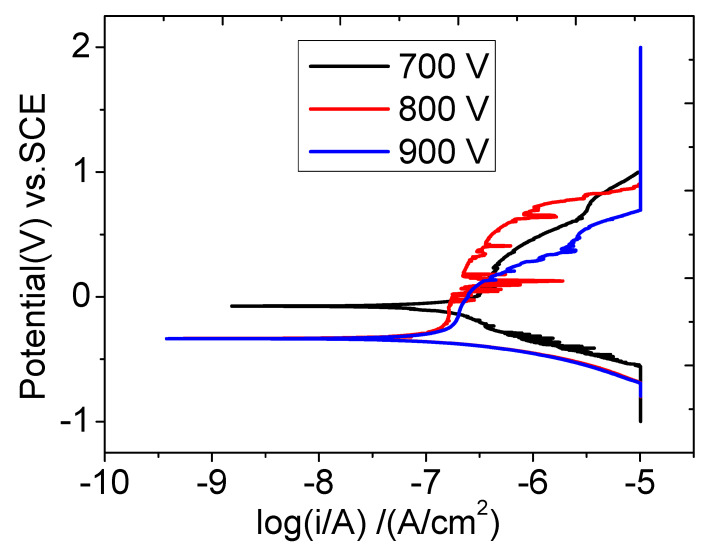
Polarization curves of Ti thin films deposited via HPPMS under different trigger voltages.

The corrosion resistance of Ti films in NaCl solution (3.5%wt) at the different voltages was studied using a potential dynamic polarization test. The corrosion resistance of Ti film was evaluated according to the self-corrosion potential and self-corrosion current density [25]. Figure 12 shows the polarization curve of Ti film prepared at the different voltages.

In Figure 12, according to the polarization curve extrapolation method, the self-corrosion potential increased significantly at 700 V, because the self-corrosion current density is the smallest at 700 V, and the lower the degree of ionization, the slower the corrosion rate of the material. At 900 V, the higher the degree of ionization, the faster the corrosion of the material, which may be because at a high degree of ionization, the substrate bombardment is too high, the stress is reduced, and the film is not dense, affecting the corrosion resistance of the film.

## 4. Discussion and Conclusions

In this work, Ti films were prepared via HPPMS deposition performed by changing the trigger voltage of the target. The plasma discharge characteristics, film structure, mechanical properties, and corrosion resistance of Ti were systematically studied. The main conclusions were as follows:

1. As the trigger voltage of the target increased from 700 V to 900 V, the peak current also increased. The ion–atom ratio near the Ti substrate gradually increased with the increase in the sputtering voltage, and the ion–atom ratio of Ti increased from 30% to 48%.
2. As the trigger voltage continued to increase to 900 V, it could be seen that the surface of the titanium film had obvious particles, indicating that the grain size of the film continues to increase with the increase in the trigger voltage.
3. With the increase in trigger voltage, the Ti ion–atom ratio increased, and the residual compressive stress generated by the Ti film changed to tensile stress and then to low compressive stress. As the voltage increased, the hardness and elastic modulus of the film were reduced, and the film was shown to not be wear-resistant. When the voltage was 700 V, the self-corrosion potential of the film was the largest, as was the corrosion resistance.

## Figures and Tables

**Table 1:** Deposition parameters of the Ti films.

Sample	Trigger Voltage/V	Peak Current/A	Average Power/w	Peak Power/kW	Frequency/Hz	Pulse Width/μs	Argon Flow/ (sccm)	Working Pressure/ Pa	Deposition Time/ min	Thickness/ nm
Ti-700	700	60	496	24			50		20	386.4 ± 16
Ti-800	800	100	1037	40	400	100		0.4		418.2 ± 20
Ti-900	900	120	1346	48						712.4 ± 59

## Data Availability

Data are contained within the article.
